# Dispositional mindfulness, affect and tobacco dependence among treatment naive cigarette smokers in Brazil

**DOI:** 10.18332/tid/105846

**Published:** 2019-04-08

**Authors:** Isabel Weiss de Souza, Elisa Harumi Kozasa, Luane A. Rabello, Beatriz Mattozo, Sarah Bowen, Kimber P. Richter, Laisa Marcorela Andreoli Sartes, Ana Regina Noto

**Affiliations:** 1 Departamento de Psicobiologia, Universidade Federal de São Paulo, São Paulo, Brazil; 2 Hospital Israelita Albert Einstein, São Paulo, Brazil; 3 Departamento de Psicologia, Universidade Federal de Juiz de Fora, Juiz de Fora, Brazil; 4 School of Graduate Psychology, Pacific University, Hillsboro, United States; 5 Department of Preventive Medicine and Public Health, School of Medicine, University of Kansas, Kansas City, United States

**Keywords:** smoking cessation, health behavior, mindfulness, tobacco use disorder

## Abstract

**INTRODUCTION:**

The current study examined associations between affective and smoking-related factors, and dispositional mindfulness among smokers seeking enrollment in a cessation program in Brazil.

**METHODS:**

Participants were first-time treatment seeking adult smokers (N=90) on a waiting list for a government-sponsored cessation program. Pearson’s bivariate correlations assessed relationships between the primary outcome variable (dispositional mindfulness) and each explanatory variable (nicotine dependence, depression, anxiety, and state positive/negative affect). Linear regression analyses evaluated the unique contribution of each explanatory variable when controlling for the others.

**RESULTS:**

The sample (N=90) was predominantly female (n=71) and most (79%) had 11 or fewer years of education. In the final regression model, a total of 36.2% of the variance in dispositional mindfulness was accounted for by positive affect (B=0.81, p<0.001), negative affect (B= -0.44, p=0.02), and level of nicotine dependence (B=1.48, p=0.007).

**CONCLUSIONS:**

Positive and negative affect, as well as nicotine dependence, account for a significant and sizable amount of the variance in dispositional mindfulness. Future mindfulness interventions for smoking cessation should be designed to address individual differences in affect, as well as nicotine dependence, in order to better tailor treatment to address baseline differences in mindfulness.

## INTRODUCTION

In spite of substantial investment in tobacco use prevention and treatment by health systems worldwide, tobacco use and tobacco-related illness remain critical public health issues. Globally, 12% of all deaths among adults aged 30 years and older are attributable to tobacco use, with the highest proportion of deaths in the developing countries of the American and European regions^1,2^.

Policies to control tobacco use are a very important component of the World Health Organization (WHO) response to the epidemic of cardiovascular diseases, cancers, chronic obstructed pulmonary disease and diabetes^2^. Strategies to promote and assist smoking cessation include fiscal measures, legislation and health-care interventions^3^.

The WHO Framework Convention on Tobacco Control (FCTC) included evidence-based tobacco dependence treatment guidelines to promote cessation^4^. A combination of cessation medications and behavioral counseling is the most effective regimen for treating tobacco dependence^5,6^. However, the substantial rates of relapse (40–70%) following smoking cessation treatment indicate a clear need for further advances in relapse prevention^7^.

In Brazil, strategic actions with a multidisciplinary approach targeted at prevention and treatment were adopted in 1988, including broad access to services that provide quality pharmacological and behavioral treatment, following international standards of good practice^8^. As a result, Brazil has seen a 50% reduction in the number of smokers over the last 20 years^9^. However, advances in treatment approaches are still needed.

Factors related to emotional regulation and stress are strongly associated with smoking relapse^10^. Cognitive-behavioral therapy is now including mindfulness-based approaches as a tool to promote psychological well-being and to target cognitive reactivity^11^. One recent review study indicates that mindfulness-based interventions are a skillful tool that mediate cognitive and behavioral automaticity, liberating the individual from the consequent suffering of dysregulation underlying addiction^12^.

Kober et al.^11^ conducted a study in which they administered a stress provocation during functional magnetic resonance imaging (fMRI) to 23 participants in a randomized controlled trial of mindfulness training (MT, N=11) versus cognitive-behavioral treatment (CBT, N=12). They found that lower neural reactivity in the amygdala and insula were associated with greater smoking reduction after treatment and at 3 months post-treatment follow-up in those who underwent MT compared to participants in CBT. These results suggest that MT treatment can potentially aid in cessation via reduction in stress reactivity.

Mindfulness-based treatment is a promising new approach for relapse prevention for smoking cessation^13,14^. Dispositional mindfulness is an individual’s capacity to observe and be aware of what is happening at the present moment, and is an inherent and modifiable trait present to some extent in everyone^15^. Among treatment-engaged smokers, higher levels of dispositional mindfulness have been shown to be associated with lower levels of dependence, higher likelihood of early abstinence, quicker recovery from abstinence after relapse, and longer maintenance of abstinence. These effects are partially explained by the mediating processes of negative emotional experiences and affect regulation^16^. Training in mindfulness is associated with emotional regulation, abstinence, and recovery following a smoking lapse^13,17,18^.

There are several possible explanations for the relationships observed between mindfulness, emotion regulation and smoking. Relationships between depression, craving and relapse may reflect the significant challenges involved in coping with the aversive internal experiences that often arise when attempting to stop any addictive behavior. Internal stressors may elicit the conditioned response of a strong desire for smoking (craving), ultimately leading to relapse^19,20^. Similarly, smokers often experience anxiety, and have difficulty observing their cognitive, affective and physical experience when feeling anxious^21^. This often results in an ‘automatic’ and habitual coping response, such as smoking^21^. Affect and craving, therefore, are critical factors in the chains of positive and negative reinforcement patterns in smokers^19,22^.

In order to understand how mindfulness training can assist with tobacco cessation, it is important to more fully understand how mindfulness relates to nicotine dependence, anxiety, depression, positive and negative affect, and how these factors interact. The objective of the present study was to evaluate the relationships among these factors. We hypothesized that negative affect, nicotine dependence, depression and anxiety would correlate negatively with dispositional mindfulness, while positive affect would correlate directly with mindfulness. We also conducted an exploratory analysis to determine the predictive potential of these factors when including the other factors in the same model. The present study is the first in Brazil to evaluate relationships between affect, dispositional mindfulness and smoking.

## METHODS

### Participants

The present study is a cross-sectional study based on the analysis of baseline data for an intervention trial evaluating the effects of mindfulness-based relapse prevention as an adjunct to tobacco cessation treatment. Because cessation medications were part of the intervention, enrollment criteria had to ensure that participants met Brazilian National Guidelines for cessation medication use, which include: a) smoking 10 or more cigarettes per day, and b) having a Fagerström Test for Nicotine Dependence (FTND) score of four or greater.

We contacted 176 individuals, age 18 years or older, from a waiting list for outpatient tobacco treatment at a municipal service for the control, prevention and treatment of tobacco use to assess interest in and eligibility for participation in the present study. Brief telephone screening assessed the following eligibility criteria: 1) Portuguese literacy, 2) physical and mental health adequate for participation in the study, and 3) smoking 10 cigarettes or more at the time of the phone call. A brief structured interview was developed by the researcher and applied in this contact asking whether potential participants were undergoing current or recent treatment for chronic diseases (depression, bipolar disorder, cancer, addiction to other drugs) and had at any prior time received treatment for smoking cessation. Those who answered affirmatively to one or both were excluded from the study. We excluded people who had received prior treatment because our sample size was somewhat small and we could not be sure that the number of people receiving prior treatment would be evenly distributed by randomization. To avoid potential confounding factors in the analyses, we decided to exclude those with prior treatment experience. In total, 116 of the 176 patients met initial criteria for study enrollment.

Out of the 116 eligible participants, 13 did not meet subsequent eligibility criteria assessed in person. Seven were unable to fill out the assessments, five presented comorbidities severe enough to limit study participation, and one scored below four on the FTND. Of the remaining 103 participants, although all reported that they were literate, 13 had substantial difficulties in understanding and interpreting the scales and were excluded. Only 90 completed all baseline assessments required for the present analyses.

### Instruments

Study measures were self-report and administered in a group setting. The FTND is a 6-item self-report measure of cigarette dependence, which yields a total score ranging from 0 to 10. It was previously translated into Portuguese and validated in Brazil and demonstrated a moderate level of internal consistency (α=0.64) and very good reliability evaluated by the test-retest (r=0.92)^23^.

The Hospital Scale of Anxiety and Depression (HAD) has been validated in Brazil and consists of 14 items assessing affective and cognitive symptoms, with subscales for anxiety and depression. For the cutoff points of 8/9, sensitivity and specificity were 93.7% and 72.6% for anxiety, respectively, and 84.6% and 90.3% for depression^24^.

The 39-item Five Facet Mindfulness Questionnaire-Brazilian Version (FFMQ-BR) uses a multidimensional approach to evaluate seven factors related to mindfulness on a 5-point Likert scale. The FFMQBR consists of seven factors, all of which demonstrate good internal consistency^25^. Cronbach’s alpha of the total scale was 0.81. We used the total score in this study to access the general level of mindfulness of the participants^25^.

The Positive and Negative Affect Schedule (PANAS) evaluates negative and positive affect in the past week via 20 items and has been validated for use in Brazil^22^. Reliability analysis revealed high internal consistencies for both the negative and positive affect scales (Cronbach’s alpha = 0.87 and 0.88, respectively)^26^.

### Procedures

All procedures were approved by The Committee of Ethics in Research of the Federal University of Sao Paulo (Unifesp) (CAAE 01949612.4.0000.5505). Participants were recruited between March 2013 and March 2015.

Participants on a waiting list for treatment were contacted by phone and invited to start the standard treatment provided by SECOPTT (the tobacco treatment service of the City of Juiz de Fora). The standard initial treatment followed the Brazilian Ministry of Health protocol for smoking cessation.

The participants who reported to SECOPTT for the first meeting were invited to participate in the present study. They were informed that they were free to either participate in the study or to decline and would receive the standard treatment irrespective of whether they agreed to participate in the study. They were also assured anonymity. Those who chose to participate signed an informed consent form and completed the baseline questionnaires, with assistance from research staff as needed.

### Data analysis

Paper surveys were double entered into Google Forms, compared for discrepancies, and corrected by consulting the original paper forms. The final data were exported to the statistics package R (R Core Team 2014) using the graphic interface RStudio (RStudio Team 2015). We performed the data analyses using the following packages: foreign, psych, car, gvlma, QuantPsyc, Hmisc and MASS. Final study results and scripts are available in the Rpubs platform and can be accessed at http://rpubs.com/leomartinsjf/mindtobacco. In the interest of reproducibility of the study, study procedures and general results are available to the public at https://github.com/crepeia/mindtobacco.

We first characterized the sample using descriptive statistics, then performed Pearson’s bivariate correlation analyses, creating a matrix of correlations between the outcome variable (dispositional mindfulness) and each predictor variable present in our hypotheses. We then evaluated several multiple linear regression models to assess the explanatory predictive contribution of each of the predictor variables when controlling for the others. In order to identify a parsimonious model, after the initial model we retained variables that had a value of p<0.10 as predictor variables of dispositional mindfulness^19^. We used several diagnostic tests to evaluate each model, including multi-collinearity, nonlinearity, non-independence of errors, non-constant error variance, and non-normality of residuals.

## RESULTS

Participants were predominantly female (n=71), and only 21.2% had 11 or more years of education. All predictor variables were significantly correlated with dispositional mindfulness (p≤0.01; Pearson Bivariate Test) (Table 1). As hypothesized, variables negatively associated with the FFMQ-BR included nicotine dependence (r= -0.31), anxiety (r= -0.33), depression (r= -0.46), and negative affect (r= -0.37). Positive affect correlated positively with the FFMQBR (r=0.49). Predictor variables were significantly correlated with one another, except for nicotine dependence and depression (r=0.19, p>0.08), positive affect (r= -0.06, p=0.58) and negative affect (r= -0.19, p=0.07).

**Table 1:** Matrix of Pearson’s bivariate correlation – dispositional mindfulness and explanatory variables of the study (n=90)

*Variables*	*1*	*2*	*3*	*4*	*5*	*6*
1 Mindfulness	M=121.5 DP=13.37	-0.31**	-0.33**	-0.46**	0.49**	-0.37**
2 Fagerström	<0.01	M=6.4 DP=2.22	0.34**	0.19	-0.06	0.19
3 Anxiety	<0.01	<0.01	M=11.4 DP=3.87	0.62*	-0.28*	0.59**
4 Depression	<0.01	0.08	0.01	M=8.6 DP=4.19	<0.01	0.08
5 Positive affect	<0.01	0.58	0.01	<0.01	M=25.3 DP=6.93	-0.27*
6 Negative affect	<0.01	0.07	<0.01	<0.01	0.01	M=20.9 DP=6.37

* p=0.01,

** p<0.01. Pearson’s r values above and p values below the diagonal.

The initial model, presented in Table 2, accounted for 36.6% of the variance in the scores of the FFMQBR (ANOVA: F(5, 84)=9.703, p<0.001). FTND scores were negatively associated with FFMQ-BR scores (B= -1.48, p=0.001), as opposed to the scores of positive affects (B= 0.72, p<0.001). Negative affect was marginally significant (p=0.10) and was thus included in the next phase of the modeling. Anxiety (p=0.73) and depression (p=0.45) were not statistically significant predictors and were thus removed from the next phase (Table 3).

**Table 2:** Coefficients of the multivariate general regression model including all predictor variables (N=90)

*Initial model*	*B*	*SE*	*B*	*p*
Constant	122.30	8.44		<0.001*
Fagerström	-1.48	0.56	-0.24	0.01*
Anxiety	0.14	0.43	-0.04	0.73
Depression	-0.35	0.45	-0.11	0.45
Negative Affect	-0.39	0.23	-0.19	0.10*
Positive Affect	0.72	0.22	0.37	0.001*

ANOVA F (5,84)=9.703, p<0.001, - R2 adjusted=36.6%.

* p<0.10.

**Table 3:** Coefficients of the multivariate general regression model including the variables selected with p<0.10 in the general model (N=90 )

*Final model*	*B*	*SE*	*B*	*p*
Constant	119.4	7.22		<0.001**
Fagerström	-1.48	0.53	-0.24	0.007**
Positive affect	0.81	0.17	0.42	<0.001**
Negative affect	-0.44	0.19	-0.21	0.02**

ANOVA F (3,86)=16.25, p<0.001, - R2 adjusted=36.2%.

** p<0.05.

The final model, presented in Table 3, accounted for 36.2% of the variance in the scores of the FFMQBR (ANOVA: F(3,86)=16.25, p<0.001; p<0.05). Negatively associated with FFMQ-BR were FTND scores (B= -1.48, p=0.007) and negative affect scores (B= -0.44, p=0.02). Conversely, positive affect was positively associated with FFMQ-BR (B=0.81, p<0.001) (Table 3).

## DISCUSSION

The results supported our study hypotheses: negative affect, nicotine dependence, depression and anxiety were significantly inversely related to dispositional mindfulness, and positive affect was significantly positively associated with dispositional mindfulness. Furthermore, positive affect was the single strongest predictor of dispositional mindfulness.

Previous research has evaluated associations between mindfulness and avoidance of aversive experiences (such as those associated with tobacco withdrawal). Specifically, studies have explored the associations between mindfulness and negative reinforcement^19,27^, as well as negative affect and nicotine dependence^10,22^ and stress reactivity and smoking^11,28^. These and negative affect studies found that lower levels of mindfulness were significantly associated with vulnerability to relapse in smokers attempting to quit^10^. The current study adds to this literature by identifying significant inverse associations between dispositional mindfulness and both negative affect and nicotine dependence, and a significant direct association between dispositional mindfulness and positive affect in a treatment-naïve population.

These results also inform the processes underlying mindfulness-based treatment for smoking cessation. For example, our findings suggest that treatments targeting emotional and behavioral regulation, such as mindfulness training, could enhance smokers’ ability to quit and maintain abstinence^29^.

To date, the quality of effectiveness trials is mixed. A recent meta-analysis by Maglione et al.^14^ analyzed 10 randomized clinical trials comparing mindfulness-based interventions with active control groups for smoking cessation, tobacco reduction and/or reduction of nicotine craving. The meta-analysis found that the effects of mindfulness training on smoking cessation did not differ significantly from control interventions. That analysis, however, underscored the need for higher quality studies with larger samples, as only one study^14^ (N=412) was adequately powered and had a strong design.

Another way to enhance future research is to create more targeted interventions, based on proposed mechanisms of action. Some studies suggest that treatments should focus on the acceptance or reinterpretation of internal triggers to lessen their likelihood of leading to adverse behavioral reactions, such as relapse. Relapse is sometimes attributed to low levels of tolerance to discomfort, including negative affect, a strong predictor of relapse to smoking^19^. In the present study, positive and negative affect accounted for more variance than anxiety and depression in levels of dispositional mindfulness; positive and negative affect were also strongly associated with nicotine dependence. Hence, relapse prevention might be an especially salient target for mindfulness interventions.

Previous studies support integration of mindfulness-based approaches for other substance use disorders^20,30-33^. Mindfulness-based relapse prevention as part of Cognitive Behavioral Therapy for substance use disorders is one such approach that is designed to improve attentional skills related to observing emotions and thoughts^34^. The practice of mindfulness does not contradict or prohibit CBT-based action plans designed to change smoking behaviors. Rather, mindfulness practice teaches observation of the mental and emotional output arising from one’s memory network^31,35^, thereby helping smokers to better manage ‘internal’ triggers.

### Limitations

The study has a number of limitations. We used a cross-sectional design, thus it is not appropriate to infer causality based on the results. For example, nicotine dependence may affect levels of affect and mindfulness, versus affect and mindfulness being predictors of dependence. Further large-scale longitudinal studies are needed to better understand the potential of mindfulness training for promoting abstinence. Another limitation is that the study sample was limited to treatment-seekers, predominantly female with minimal education level, and thus may not represent all smokers.

## CONCLUSIONS

The results of the present study lay the groundwork for further evaluation of mindfulness as a relapse prevention intervention for smoking cessation and identify affect as a critical potential moderator of treatment effects. The results also provide a basis for future studies in Brazil that aim at advances in the clinical approach to smokers, in a country where the standard treatment is cognitive-behavioral.
